# Supplementary material for: Are carbon emissions trading and green financial instruments synergistic? -Comprehensive quantitative research based on content analysis
Source: PLoS One. 2024 Mar 7;19(3):e0298601. doi: 10.1371/journal.pone.0298601 (PMC10919601; doi:10.1371/journal.pone.0298601)
Supplement: S1 Data — (ZIP) [file pone.0298601.s001.zip › Data/地级市Z_F工作报告.zip!/╘╞─╧/╙±╧¬╩╨/└┤╫╘╣1⁄2╓┌║┼í╛┬φ┐╦╩2╛▌═°í┐_2016.docx]

各位代表：

　　我代表市人民政府，向大会报告政府工作，请连同《玉溪市国民经济和社会发展第十三个五年规划纲要（草案）》一并审议，请市政协委员和列席人员提出意见。

　　一、“十二五”工作回顾

　　“十二五”以来，在省委、省政府和市委的坚强领导下，在市人大和市政协的监督支持下，市人民政府团结和依靠全市各族人民，坚持发展第一要务，主动适应经济发展新常态，集中精力稳增长、调结构、促改革、惠民生、保稳定，协调推进经济、社会、文化、生态各项建设，努力克服金融危机、多年连旱、经济下行等诸多挑战，经济社会平稳健康发展，“十二五”规划圆满收官，为“十三五”发展打下了坚实基础。

　　经济实力大幅提升。全市上下聚焦稳增长，合力打好园区经济、民营经济、县域经济三大战役，出台一系列政策措施，促进经济增长。生产总值突破千亿元，由2010年736.4亿元增加到2015年1245.7亿元，年均增长10.2%；人均生产总值由32068元增加到52877元，年均增长9.7%；财政总收入由304.4亿元增加到506.3亿元，年均增长10.7%；一般公共预算收入由64.7亿元增加到124.8亿元，年均增长14%；规模以上固定资产投资由211.9亿元增加到667.6亿元，年均增长25.8%；社会消费品零售总额由141.5亿元增加到291.4亿元，年均增长15.6%。县域经济发展势头强劲，新平县生产总值突破百亿，易门县发展速度居全省前列。玉溪高新区升格为国家高新区。工业园区建设发展加快，增加值年均增长7.4%。

　　经济结构不断优化。全力转方式调结构，全面实施产业建设年三年行动计划，巩固提升传统支柱产业，积极培育战略性新兴产业，促进结构优化、效益提升。三次产业结构由9.4∶62.2∶28.4调整为10.2∶55.6∶34.2，培育了褚橙、云菜、猫哆哩等品牌，农业增加值突破百亿，年均增长6.9%，2014年“三农”发展综合考评名列全省第一；红塔集团为稳增长作出重要贡献，装备制造业快速发展，工业增加值年均增长10.4%；旅游总收入突破百亿，第三产业增加值年均增长9.8%。非公经济比重由32.1%提高到34%，非烟经济比重由62.7%提高到66.2%。

　　改革开放深入推进。紧盯重点领域、关键环节改革攻坚和对外开放，突出问题导向，着力优化发展环境，激发创新创造活力。推进行政审批制度改革，下放土地供应、环境影响评价审批权限，行政审批事项取消133项、下放158项、调整141项，非行政许可审批事项全部取消。配合云南中烟两统一两整合改革，红塔银行即将挂牌，市属投融资公司“7变3”改革重组完成。商事制度改革激发了市场活力，民营企业增加6.5万户。殡葬改革取得重大突破，火化区火化率达100%、骨灰100%进公墓。创造了医改“玉溪模式”，公立医院改革列为国家试点。园区实体化、财税体制、农村综合、事业单位分类、公务用车、户籍制度、文化体制等改革稳步推进。努力扩大对外开放，累计使用市外国内资金1762亿元、年均增长47.5%，进出口总额44.2亿美元、年均增长45.6%。

　　基础设施日益完善。着力打基础破瓶颈，打好“五网”建设攻坚战。玉蒙铁路建成运营，昆玉铁路扩能改造即将完成，玉磨铁路、高铁新城开工建设。新三、易峨高等5条高等级公路和呈澄、石红高速建成通车，晋红高速建设进展顺利，玉临、江通、武易高速开工，新增高等级公路291公里，改扩建农村公路2808公里。江川护林防火直升机场开工建设。新增110千伏以上输变电站27座，风电开发实现零突破。建设输油管道178公里、天然气管道118公里，推广天然气用户2万户。行政村光缆通达率100%，宽带用户普及率48.8%，4G移动通信网络实现乡镇全覆盖。改造中低产田地99万亩。新建扩建水源工程10件，病险水库除险加固341件，解决了39.7万人饮水安全问题。

　　生态环境持续改善。突出以“三湖”保护治理为重点的生态建设和环境保护，出台实施争当全省生态文明建设排头兵的意见，落实环境保护“一岗双责”，生态建设迈出新步伐。坚持“五个坚定不移”，投资61.7亿元实施79个“三湖”保护治理项目，“十二五”水污染综合防治规划项目基本完成，332个村环境综合整治、31条主要入湖河道治理取得实效，抚仙湖水质总体保持Ⅰ类，星云湖、杞麓湖水质有所改善，东风水库、飞井海水库环境综合整治成效明显。严格管控抚仙湖周边开发，退出开发项目11个、减少规划用地7.2万亩。抚仙湖列为国家首批湖泊生态环境保护试点，沿湖3县纳入国家重点生态功能区转移支付范围，抚仙湖、杞麓湖列入国家湿地公园试点。完成营造林221万亩，种植核桃97万亩、竹子18万亩，森林覆盖率由54.2%提高到57%。开发整理土地24.9万亩，治理水土流失面积930平方公里。淘汰落后产能445万吨，取缔黄标车2万辆，全面开展PM2.5监测和治理，空气质量明显好转。

　　城乡建设步伐加快。统筹城乡协调发展，着力推进以人为核心的新型城镇化，城乡面貌明显改观。城市建成区面积72.8平方公里，城镇化率达46.9%。完成中心城区玉溪大河二期、东近面山休闲公园、平战结合人防工程等重大项目建设，新天地、红星国际等城市综合体有序推进。城乡环境综合整治效果显著，拆除临违建筑266.8万平方米、绿化43万平方米。荣获国家卫生城市称号并通过复检，国家园林城市得到巩固，江川撤县设区获国务院批准。建设城镇保障性住房7.6万套，解决了近6万人住房困难。完成农村危房改造8.6万户。实施38个美丽乡镇规划建设，3个县成为国家生态示范区，2个县创建为国家园林县城，13个集镇列为省级特色小镇。城镇生活污水处理率达85%，城市生活垃圾无害化处理率达95.4%，农村生活污水处理率达37.7%。

　　人民生活显著提高。围绕保就业促增收，投入691亿元，着力保障和改善民生。新增城镇就业11.3万人，扶持创业7.1万人，转移农村劳动力17.6万人，城镇登记失业率控制在3.5%以内。城乡居民社会保障水平不断提高，新农合人均筹资标准由140元提高到470元。实施扶贫攻坚项目1065个，26.8万人实现脱贫。落实移民安置和后期扶持政策，溪洛渡水电站移民化念安置工作顺利完成。城乡居民人均可支配收入分别达2.96万元、1.1万元，年均分别增长12%、14.1%。城镇每百户拥有汽车50.4辆，城镇居民人均住房面积58.6平方米，每千人拥有执业医师2.1人、病床4.4张，平均预期寿命76.1岁。

　　社会事业全面进步。强化基本公共服务供给、市民素质提升、社会和谐稳定等工作，加大社会事业投入，促进人的全面发展。建成美丽校园85所，消除D级危房81.8万平方米，新建改造校舍153.8万平方米，“全面改薄”顺利推进，“三免一补”实现全覆盖，人均受教育年限达8.5年。实施省级以上科技项目546项，专利申请和授权量居全省第二。中国聂耳音乐（合唱）周、玉溪灯会等影响力不断扩大，澄江化石地列入世界自然遗产，滇剧《水莽草》荣获“五个一工程”奖。城乡医疗卫生体系不断完善，县级医院托管乡镇卫生院稳步推进。全民健身广泛开展，成功举办各类体育赛事，奥运金牌实现零突破。连续三届获全国“双拥”模范城称号。民族团结进步示范区建设取得阶段性成效。公共安全管理全面加强，连续多年未发生重特大安全生产事故。市县乡三级视频接访系统建成运行，开展严打暴恐专项行动，强化禁毒防艾工作，荣获全国社会管理综合治理优秀市和全国法治建设先进市称号。妇女儿童、老龄、残疾人、红十字、关心下一代等事业健康发展，防灾减灾、外事侨台、供销、档案、气象等工作取得新成绩。

　　刚刚过去的2015年，市人民政府深入贯彻习近平总书记系列重要讲话和考察云南重要讲话精神，全面落实李纪恒书记、陈豪省长在玉溪调研重要讲话精神，严格执行市委“七位一体”工作机制和“八要”工作方案等各项决策部署，把稳增长作为经济工作的重中之重，出台26条具体措施，促进经济平稳健康发展，主要目标任务基本完成。全市生产总值增长8.5%、低于人代会目标1.5个百分点，社会消费品零售总额增长12.5%、低0.5个百分点，城乡居民人均可支配收入分别增长8.8%和10.1%，分别低2.2个和2.9个百分点；一般公共预算收入增长9.9%，规模以上固定资产投资增长30.4%，居民消费价格总水平上涨2%，城镇登记失业率3.5%，万元生产总值能耗下降8%。

一年来，我们重点做了6项工作：

　　全力以赴抓产业。大力发展高原特色农业，粮食总产“十连增”，烟叶种植收购任务圆满完成、烟叶税增长9.1%，蔬菜、水果等农产品出口创汇14.8亿美元，畜牧业产值增长6.3%，精品庄园、农业庄园建设稳步推进，新增市级以上龙头企业29户、农民专业合作社64个。设立10亿元产业发展基金和2.5亿元工商企业信贷扶持资金，合和集团落户玉溪，卷烟及配套产业增加值增长3.5%；年产400台风力发电机、80万辆电动车生产线建成投产，奇瑞万达新能源汽车项目成功落地，通用航空产业发展战略合作协议成功签约，装备制造、生物医药及食品、新能源新材料及节能环保等产业实现新发展。落实扩销促产、清费降税等优惠政策，3户企业在新三板上市。信息产业发展实现新突破，成功举办第二届中国电子商务园区峰会，华为玉溪云计算数据中心主体工程完工，互联网产业园、磷资源交易中心、淘宝“特色中国?玉溪馆”挂牌运营，引进亿赞普、华唐教育、融创天下等知名企业，与腾讯、阿里巴巴合作不断深入，农村电商“千县万村”试点县通海建成50个村级淘宝服务站，启动全国电子商务进农村综合示范工作，“互联网+教育、卫生、纪检监察、城市服务、扶贫、组织”成效初显。仙湖时光栈道、哀牢古镇、元江红河谷热海等项目进展顺利，启动6个特色旅游村建设，希尔顿酒店建成营业，旅游总收入增长16%。

　　千方百计增投资。投入3.3亿元前期工作经费，设立玉溪中介超市，明确项目、进度、问题、责任4个清单，现场推进重大项目建设，“四个一百”项目完成投资480亿元，房地产业、交通运输业、工业、农业投资分别增长38.6%、41.4%、28%、108.9%。成功举办百名客商、百户国企进玉溪洽谈会和北京顺义区招商推介会，富滇、中信、福建三禾资本落地发展，引进省外国内资金475亿元，增长23.4%。强化资金要素保障，争取上级资金242亿元、增长1.1倍，其中省级转贷债券资金132亿元；与省农发行、省建行签订战略合作协议，全市新增贷款69亿元。创新投融资方式，上报省级PPP项目57个，争取国家专项建设基金项目27个、资金64.1亿元。

　　统筹协调建城乡。“四规合一”试点稳步推进，“六城同创”全面启动，省级节水型城市通过验收，玉溪列为国家智慧城市试点。中心城区重点项目进展顺利，聂耳东路、城南客运站、梅园立交拆除重建主体等工程完工，武警支队迁建主体工程竣工，城市生活垃圾焚烧项目、第二污水处理厂开工建设。20个棚户区项目全部开工。启动集镇“一水两污”项目37个，新建污水管网67公里。深入开展城乡人居环境综合整治三年行动计划，实施18个美丽乡镇和47个示范村、393个整治村建设，农村改厕500座。

　　坚定不移保生态。出台生态文明体制改革实施方案。抚仙湖径流区实现统一托管，北岸生态湿地搬迁一期安置房封顶，生态调蓄带一期主体工程完工，华宁大龙潭引水工程正式通水。建成8.4公里星云湖生态调蓄带，杞麓湖周边废弃菜叶资源化利用效益显现。关闭非煤矿山25座，新增耕地1.1万亩。完成污染减排项目138个。县城空气质量逐步好转，中心城区空气一级天数增加57天、超标天数减少5天。

　　主攻短板惠民生。盘活财政存量资金5.5亿元用于民生项目，10件实事圆满完成。推动大众创业、万众创新，建成大学生文化教育创业园、青年创业园，就业创业工作得到加强。完成机关事业单位工资收入分配制度改革，120万人参加城乡居民基本养老保险，发行“金融社保卡”100万张。全面开展“挂包帮”“转走访”，完成2个整乡、34个整村推进扶贫项目。建成农村敬老院6个、居家养老服务中心40个、农村幸福院70个。美丽校园建设成为全省样板，义务教育全面实现学区化办学，“三免一补”、营养改善计划、困难学生资助等工作成效明显。市急救中心、市儿童医院、市医院改扩建等项目顺利推进，完成2个乡镇卫生院、15个村卫生室标准化建设，区域卫生信息平台通过国家级成熟度测评，公立医院取消药品加成，DRGs付费制度改革试点工作得到国家有关部委肯定，列为全国精神卫生综合管理试点市。承办纪念世界遗产30周年、联合国教科文组织70周年暨建立国家公园体制专题研讨会，“三台”合并、“四个一”创拍等工作快速推进。第十届全国民运会上荣获2枚金牌。完成全国1%人口抽样调查。

依法行政提效能。“四五”依法治市顺利推进，“六五”普法圆满完成，平安玉溪、法治玉溪建设有序开展，社会大局和谐稳定。严格执行市委重大决策，自觉接受人大法律监督、工作监督和政协民主监督，人大代表建议和政协提案解决率分别达49.4%、46.4%。公布市级部门权力清单和责任清单，开通行政审批和电子监察系统，行政审批网上服务大厅建成运行，四级政务服务体系基本建成。出台玉溪市重大行政决策程序规定，组织重大决策听证45项，政务信息公开深入推进。市、县区政府及其工作部门法律顾问实现全覆盖。开展“三严三实”和“忠诚干净担当”专题教育，严格执行中央八项规定精神，“三公”经费支出下降22%。落实党风廉政建设“一岗双责”，强化审计监督，深入开展反腐败斗争，纠风治乱取得实效。

各位代表，“十二五”以来的五年，我市经济社会发展取得了巨大成就，成绩来之不易。这是市委科学决策、坚强领导的结果，是市人大、市政协和各民主党派、工商联、社会各界监督支持的结果，是中央、省驻玉单位、军警部队关心帮助的结果，是各位老领导、离退休老同志热情支持的结果，是全市广大干部群众勠力同心、顽强拼搏的结果。在此，我代表市人民政府，向大家表示崇高的敬意和衷心的感谢！

　　在肯定成绩的同时，我们也深刻认识到，发展中还存在很多困难和问题：一是支柱产业单一，烟草和矿冶两大传统产业增长动力逐步减弱，新兴产业培育滞后，发展方式粗放，创新能力不强，质量效益不高，部分行业产能过剩，企业生产经营困难，财政收支矛盾突出，经济运行潜在风险增多，稳增长压力大；二是城乡之间、区域之间发展不平衡，城镇化水平低，特别是山区、少数民族地区基础设施薄弱、脱贫攻坚任务重、群众就业增收难，教育、医疗、文化等方面仍有不少薄弱环节，保民生压力大；三是土地、空气、水等资源环境约束趋紧，“三湖”流域水污染防治任务依然艰巨，农业农村面源污染尚未得到有效解决，生态环境保护压力大；四是政府职能转变还不到位，改革创新意识不强，缺乏引领经济新常态的办法和手段，执行力和工作效率有待提高；五是“庸懒散混”在一些地方和部门依然存在，少数公职人员为官不为、为官不勤、为官乱为，有的甚至顶风违纪、以权谋私，严重影响了党和政府的形象。这些问题，我们将高度重视，在今后的工作中认真加以解决，努力让人民群众满意。

　　二、“十三五”目标任务

　　未来五年，是我市在全省率先全面建成小康社会的决胜阶段。根据市委关于制定“十三五”规划的建议，市人民政府组织编制了《玉溪市国民经济和社会发展第十三个五年规划纲要（草案）》。指导思想是：高举中国特色社会主义伟大旗帜，以邓小平理论、“三个代表”重要思想、科学发展观为指导，深入贯彻习近平总书记系列重要讲话和考察云南重要讲话精神，全面贯彻党的十八大和十八届三中、四中、五中全会，省委九届十次、十一次、十二次全会及市委四届七次全会精神，坚持“五位一体”总体布局和“四个全面”战略布局，牢固树立创新、协调、绿色、开放、共享发展理念，坚定不移地实施生态立市、产业强市、创新活市、开放兴市战略，坚持发展第一要务，以提高发展质量和效益为中心，加快形成引领经济发展新常态的体制机制和发展方式，统筹推进经济建设、文化建设、社会建设、生态文明建设，奋力闯出一条跨越式发展的路子来，在全省率先全面建成小康社会，为谱写中国梦的玉溪篇章奠定坚实基础。主要目标建议为：生产总值年均增长9%左右，规模以上固定资产投资年均增长20%以上，一般公共预算收入年均增长5%以上，城乡居民人均可支配收入年均分别增长10%和11%以上，努力实现经济社会跨越式发展，争当全省民族团结进步示范区，争当全省生态文明建设排头兵，建设国际大通道和现代物流重要枢纽、辐射南亚东南亚的重要基地，建设生态宜居文明幸福的魅力之城。

　　实现“十三五”规划目标，要将认识新常态、适应新常态、引领新常态作为贯穿发展全局和全过程的大逻辑。把创新作为发展的强大动力，持续推动科技、制度、管理等方面的创新，靠创新调整结构、靠创新增强活力、靠创新拓展空间。把协调作为发展的内在要求，界定在滇中城市经济圈中的发展定位，促进经济与社会、农村与城市、山区与坝区互动发展，推动新型工业化、信息化、城镇化、农业现代化同步发展，增强发展整体性。把绿色作为发展的先决条件，坚持绿水青山就是金山银山，引导全社会形成尊重自然、顺应自然、保护自然的生态文明理念，严守资源消耗上限、环境质量底线、生态保护红线，推动绿色低碳循环发展。把开放作为发展的必由之路，统筹国际国内两个市场、两种资源，充分发挥区位优势，大力发展开放型经济，加强与南亚东南亚国家的深度合作，实现互利共赢，不断提高对外开放水平。把共享作为发展的本质要求，动员全市人民共同参与、共同尽力经济社会发展，持续加大民生投入，增加公共服务供给，建立更加公平更可持续的社会保障制度，让人民群众有更多获得感幸福感。我们一定要突出事关玉溪发展全局的7大重点，抓基础、调结构、转动力、补短板、防风险，确保全面建成小康社会目标顺利实现。

　　（一）集中精力培育支柱产业。坚持产业强市战略，紧扣“三区一港”发展定位和“342”产业布局，坚定不移转方式调结构，推动产业升级。巩固提升卷烟及配套、矿冶、高原特色农业3大传统支柱产业，继续发挥好“稳定器”作用。积极培育文化旅游、生物医药及食品、装备制造、现代物流4个新的支柱产业上下功夫，形成新的经济增长极。大力发展信息、新能源新材料及节能环保2个新兴产业，储备新的后续产业。创新产业扶持方式，以园区为载体优化产业布局，明确产业发展重点，推动园区特色化、产业集群化发展。力争到2020年，3大传统支柱产业增加值稳定在830亿元，4个新的支柱产业达500亿元，2个新兴产业达160亿元，销售收入超千亿元园区2个、超百亿元园区9个。

　　（二）改革创新增强发展新动力。坚持创新活市战略，发挥创新引领作用，增强改革发展动力，为实现跨越式发展提供强大动力和有力保障。深化供给侧结构性改革，从供需两端双向发力，持续推进市县政府简政放权、放管结合、优化服务，充分发挥市场在资源配置中的决定性作用，优化土地、资金、技术、劳动力等要素配置，帮助产品有市场、技术含量高的企业节本增效，让产品附加值低、资源消耗高、产能过剩的企业有序退出，扩大农田水利、道路交通、扶贫开发、教育卫生等公共产品有效供给，扶持市场前景好的健康养老、文化旅游、金融保险等服务业加快发展。实施创新驱动发展战略，强化企业创新主体地位，把科技创新的重点放在装备制造、生物医药、高原特色农业、信息等领域，引进消化一批、研发创新一批、引入高端人才带入一批。着力营造创新环境，引进和培养各类创新型人才，协调推进管理方式、商业模式、服务方式等变革，激活大众创业、万众创新。到2020年，全社会研发经费投入占GDP比重达到2.5%以上，科技进步对经济增长的贡献率达60%以上。

　　（三）打赢基础设施建设攻坚战。坚持打基础、破瓶颈、强支撑，大力推进路网、水网、能源保障网、互联网、航空网建设，构建现代基础设施体系。开通昆玉高铁，抓好玉磨铁路、2条城际铁路和10条高速公路建设，加快国省干线和农村公路建设，规划建设货运机场和通勤机场，新增高速公路450公里，实现县县通高速、50户以上自然村100%通硬化路目标。配合做好滇中引水工程，实施水保障网重点工程237件，新增供水2.58亿立方米。加快推进电源电网、燃气管道网建设，自发电量突破100亿千瓦时、天然气使用量超过5亿立方米。大力发展物联网、大数据、云计算和移动互联网，加强高速互联网出口通道建设，实现行政村通光纤、4G网络全覆盖，把玉溪建成面向南亚东南亚重要的通信枢纽和区域信息汇集中心。

　　（四）建设对外开放合作新高地。坚持开放兴市战略，以开放促发展、发展促开放，加快发展外向型经济。主动融入和服务国家“一带一路”、长江经济带发展战略，加强与长三角、珠三角地区交流合作，规划建设承接产业转移示范园区。加快综合保税区申报建设，积极争取设立海关、检验检疫机构，布局建设国际物流场站、国际贸易始发港和目的港。培育一批外向型龙头企业，建设全省最大的农产品加工出口示范区，支持优势产业和企业到境外投资发展，外贸进出口总额年均增长20%以上，将玉溪建成辐射南亚东南亚的重要基地。大力推动产业入玉、资本入玉、科技入玉、人才入玉，引进市外国内资金年均增长15%以上。

　　（五）争当生态文明建设排头兵。坚持生态立市战略，牢固树立绿色发展理念，实施“六大工程”，推动绿色低碳循环发展，加快资源节约型、环境友好型社会建设，实现永续发展。落实主体功能区规划，推动各县区依据主体功能定位加快发展，促进人口、经济、资源环境相协调。实施“三湖”水污染综合防治“十三五”规划，完成抚仙湖、星云湖一级保护区3.3万人搬迁，加强杞麓湖湿地建设，确保抚仙湖水质总体保持Ⅰ类，消灭星云湖、杞麓湖劣V类水质。全面落实大气、水、土壤污染防治行动计划，加强重金属污染防治。深入推进森林玉溪建设，实施造林绿化工程，森林覆盖率达60%以上。开展循环经济示范行动，推进水、土地、矿产等资源节约高效利用，完成省下达的节能减排约束性指标。

　　（六）协调推进城乡一体化发展。牢固树立协调发展理念，构建工农之间、城乡之间、区域之间协调发展的体制机制，科学布局山水田路产城，做到生产空间集约高效、生活空间宜居适度、生态空间山清水秀。坚持规划先行，推动“多规合一”，统筹考虑国土空间开发布局，着力解决城市规划水平不高、执行不力，乡村规划缺位、建设无序等问题。坚持以人为本，深化户籍制度改革，维护进城落户农民土地承包权、宅基地使用权、集体收益分配权，实现基本公共服务常住人口全覆盖，有序推进农业转移人口市民化；推动城乡土地、资金、劳动力等要素平等交换，教育、卫生、文化等公共资源合理配置，推动基础设施建设向农村延伸。尊重城镇发展规律，科学界定中心城区和县城空间、规模、产业，引导新型城镇化科学有序发展，提高城乡综合承载能力和治理能力，打造具有地域和民族特色、彰显自然山水的新型城镇。认真实施滇中城市经济圈一体化发展总体规划，推动昆玉一体化发展，加快“三湖”生态城市群建设，建设全省区域性中心城市。全面消除农村D级危房，保护好传统村落和名村名镇，深入推进城乡人居环境综合整治三年行动计划，改善农村人居环境，建设美丽宜居乡村。力争全市城镇化率达60%。

　　（七）织牢织密民生安全保障网。牢固树立共享发展理念，保基本、兜底线、促公平，让全市人民共享发展成果。全力以赴打赢脱贫攻坚战，实施精准扶贫、精准脱贫，确保2017年彻底消除现行标准下农村贫困人口、9个贫困乡镇和75个贫困村全部摘帽出列。完善就业创业扶持政策，实施“就业创业玉溪”行动计划，确保城镇登记失业率控制在4.5%以内。建立健全城乡统一的基本医疗保险制度，完善社会保险和社会救助体系。基本普及学前教育，均衡发展义务教育，普及高中阶段教育，构建现代职业教育体系，积极发展高等教育，到2020年劳动年龄人口平均受教育年限达到10.5年。健全完善公共卫生、医疗卫生服务体系，鼓励和发展社会办医，推进健康玉溪建设。加强公共文化体系建设，繁荣文艺事业，发展文化产业，建设文化强市。加强民族宗教工作，争当全省民族团结进步示范区。加强食品安全工作。健全公共安全保障体系，全面推进法治玉溪、平安玉溪建设，争当“平安云南示范区”，争创全国“长安杯”。

　　三、2016年主要工作

　　今年是“十三五”规划的启动之年，也是全面建成小康社会进入决胜阶段的开局之年。我们要认真贯彻党的十八届五中全会、中央经济工作会议和省委省政府的重大决策部署，全面落实市委经济工作会议确定的各项目标任务，坚定不移地实施生态立市、产业强市、创新活市、开放兴市战略，更加注重发展的质量和效益，更加注重深化改革扩大开放，更加注重统筹城乡协调发展，更加注重生态文明建设，更加注重增进人民福祉，推动经济社会持续健康发展。经济社会发展主要目标建议为：生产总值增长9%左右，规模以上固定资产投资增长25%以上，一般公共预算收入增长5%以上，社会消费品零售总额增长11%以上，城镇居民人均可支配收入增长10%以上，农村居民人均可支配收入增长11%以上，居民消费价格涨幅控制在3%以内，城镇登记失业率控制在4.3%以内，城镇化率达到49%。

实现上述目标，要突出抓好9个方面的工作：

　　（一）保持经济平稳发展

　　推进供给侧结构性改革。围绕去产能、去库存、去杠杆、降成本、补短板5大任务，利用好结构性减税、差别电价、财政金融扶持等调控手段，创造新供给，培育新需求，增强新动力。积极稳妥化解钢铁、水泥、铁合金等过剩产能，完成淘汰落后产能任务，防止新的产能过剩。出台促进房地产健康发展实施意见，建立购租并举的住房制度，促进存量商品房去库存化。落实中央、省减轻企业负担各项政策，清理中介服务，减少涉企收费，降低企业成本，提高盈利水平。努力增加基础设施、生态环保、公共服务等方面的供给，补齐发展短板。加强地方政府债务管理，改善债务结构，防范债务风险；强化金融监管，防范区域性风险。

　　突出投资关键作用。坚持重大项目市级领导联系制，强化县区、部门主体责任和投融资平台筹资建设责任，落实项目、进度、问题、责任清单，抓好省市“四个一百”重点项目建设。创新项目前期经费使用方式，建立投入退出机制，抓实抓深前期工作，增加好项目大项目储备。争取上级资金增长10%以上。放宽市场准入，推广PPP模式，撬动社会资金投入。完善国家专项建设基金3年滚动项目库，积极争取专项基金支持。依托友好城市、滇沪合作机制等平台，强化招商引资，突出精准招商，重点考核项目开工率、资金到位率、投资完成率，确保实际引进省外国内资金增长14%、外资增长5%。

强化消费基础作用。实施重点领域消费工程，落实鼓励消费政策，优化消费环境，释放消费潜力，增强消费拉动力。健全完善旅游市场，落实带薪休假政策，努力增加旅游消费。稳定汽车、教育、文化等消费，拓展信息、家政、养老、健康消费。鼓励支持企业建设电子交易平台，完善农村商业网点，推进农村电子商务“千县万村”试点县建设。扶持一批商贸流通企业，抓好专业市场、便民“农超”建设，完成5个省级、13个市级市场建设。

发挥出口促进作用。落实出口促进政策，加强农产品出口基地建设，扩大蔬菜、水果等优势产品出口规模，推动电动摩托车、新能源汽车等开拓东南亚市场。扩大资源性产品、先进技术装备、关键零部件元器件等产品进口。清理和规范进出口环节收费，鼓励支持更多企业“走出去”建立营销窗口、生产基地，开展服务外包。外贸进出口总额增长20%以上。

　　加强经济运行调节。发挥9大产业发展工作小组作用。强化经济运行分析预警和稳增长政策措施落实，搞好土地、资金和煤电油运气等协调保障，提高经济运行调节的针对性和有效性。建立土地利用年度计划指标市级统筹调配机制，清理批而未供、供而未建土地，把用地指标向大项目多、发展潜力好的地方倾斜。创新政府投资体制机制，提高产业发展基金使用效益，探索设立环保基金和城市建设基金，发挥财政资金放大效应。加强与金融机构协调合作，密切银政、银企关系，新增贷款80亿元。

　　（二）改善基础设施条件

　　加快交通设施建设。完成昆玉电气化铁路扩能改造，配合做好玉磨铁路征地拆迁工作，启动马金铺至澄江轨道交通建设。开工建设澄川、大戛高速和弥楚、永金高速玉溪段，加快玉临、晋红、江通、武易高速建设和老国道213线改造。推进江川护林防火直升机场建设，做好元江通勤机场前期工作。

　　加快水利设施建设。完成华宁大龙潭引水支管工程和58件病险水库除险加固，加快鲁布、苗茂中型和5件小（一）型水库建设，力争矣则河中型水库开工建设、筇川等4件小（一）型水库立项批复，实施农村饮水安全巩固提升工程。抓好以“三湖”流域为重点的农业高效节水减排项目实施。配合做好滇中引水工程玉溪受水区水利配套设施等项目前期工作。推进戛洒江、绿汁江、南盘江干支流河道治理。

　　加快能源设施建设。加快戛洒江一级水电站建设，抓好3个220千伏、15个110千伏输变电工程。完成3个风电场、1个光伏发电站建设，开工建设2个风电场。完成中石油玉溪配套油库、应急气源储备中心和3条天然气管道支线建设，新建燃气管网110公里，推广天然气用户1万户。

　　加快信息基础设施建设。启动宽带接入网和骨干网、城域网能力提升工程，加快“宽带乡村”、下一代广播电视接入网络、移动宽带网覆盖等重大项目建设，推进信息消费、信息惠民、智慧城市等关联项目建设。建立信息共享机制，防止重复投资。

　　（三）加强农业农村工作

　　打好脱贫攻坚战。实施“五个一批”精准脱贫，层层明确责任，逐级传导压力，扎实开展“挂包帮”“转走访”工作，动员各方力量攻坚拔寨，完成因远和平掌整乡推进，加快富良棚和铜厂整乡推进，启动2个以上整乡推进项目，完成40个整村推进任务。实施1万人易地搬迁等项目建设，确保4万贫困人口脱贫。推进移民新村建设，做好后期发展扶持工作。

　　大力发展高原特色农业。以发展开放型农业为突破口，制定具体实施意见，大力发展生态循环现代农业，推动农村三次产业融合发展，确保农业增加值增长6.5%。扶持建设一批农产品专业市场、冷储冷链物流设施、境外销售窗口，启动农产品质量安全检验检测中心、认证中心建设，组建营销集团，建设面向南亚东南亚的加工基地和集散地。开展农业生态环境提升行动计划，扶持发展一批竞争力强的优势农产品，种植烤烟57万亩、收购烟叶153万担，种植粮食160万亩、蔬菜110万亩，新增核桃8.5万亩、水果2万亩，创建3个畜禽标准化和10个循环养殖示范场。扶持发展涉农企业“小巨人”，新增5户市级龙头企业、40个农民专业合作社，建设1个省级精品庄园、10个市级庄园、50个市级家庭农场，培养新型职业农民1500人。

　　推进美丽宜居乡村建设。持续开展“百村示范、千村整治”行动，建设40个示范村，整治400个重点村。统筹推进省级重点建设村、美丽乡村示范点、民族团结进步示范村和民族特色村寨建设。加强村庄规划建设管理，制定农村宅基地管理办法，规范农民建房行为，完成农村危房改造4万户。改造中低产田地19.7万亩。深入开展城乡人居环境综合整治，新改扩建农村公路1000公里，加大公厕、照明等配套设施建设力度，完善“组保洁、村收集、镇转运、县处置”的农村生活垃圾处置体系，切实改善农民生产生活条件。

　　激发农村发展活力。深入推进农村集体建设用地使用权、土地承包经营权、宅基地使用权制度改革，流转土地3万亩，完成农村土地承包经营权确权登记颁证104万亩。扩大政策性农作物保险范围，增强农业抵抗风险能力。抓好涉农水、电等生产资料价格改革，打牢持续增收基础。做好第三次全国农业普查工作。

　　（四）推动产业转型升级

　　促进工业做优做强。加强优质烟叶原料基地建设，支持红塔集团搞好技术改造、优化产品结构，确保税利增长5%以上；强化与合和集团合作，整合配套企业，提升产业竞争力。推进矿冶产业转型升级，大力开发金属制品、精细磷化工等产品，延伸产业链，提高附加值。积极发展装备制造业，制定实施“中国制造2025”玉溪行动计划，抓好上海电气风电设备、奇瑞万达新能源汽车等项目建设，打造产业集群。加快发展新兴产业，以沃森单抗和HPV疫苗产业化、稀贵金属回收、锂电池、LED等产品为重点，加快沃森现代生物医药产业园、稀贵金属循环经济产业园等园区建设。联合国内外研发机构，集中力量实施一批重大科技专项，推进重点领域突破关键核心技术，完善以企业为主体的技术创新体系，支持企业加强科技、管理、品牌、组织、商业模式创新，新认定5户市级工程技术研究中心或重点实验室。确保工业增加值增长8%以上。

　　大力发展第三产业。落实昆玉红旅游文化产业经济带建设规划，搭建旅游开发投资平台，抓好寒武纪乐园、仙湖时光栈道等重大项目，加快红塔古镇、哀牢古镇、仙湖小镇和10个农业旅游庄园、10个旅游村建设，完善配套设施，强化市场管理，打造旅游品牌，确保旅游总收入增长10%以上。建成华为云计算数据中心，加快华唐教育大数据及服务外包产业示范基地、融创天下微总部、亿赞普大数据跨境贸易平台等项目建设，信息产业增加值增长30%。推进东南亚食品商贸仓储物流港、活发现代商贸物流园、通力物流基地等项目建设，培育壮大物流企业，加快发展物流产业。积极发展生产性服务业和生活性服务业，规划建设一批现代服务业集聚区。确保第三产业增加值增长10.5%。

　　推动产业集聚发展。明确主导产业定位，强化产业布局市级协调和招商项目预审，打造专业化特色化园区，切实解决产业布局散乱问题。推动重化工产业向大化园区转移，整合红塔区工业园区，完成4个市级园区实体化改革。加快高新区南片区退二进三和九龙片区、龙泉片区开发，推动重庆东恩产业园、中德国际生态产业园、北京顺义产业园等园中园建设，启动通用航空产业园建设。将工业园区扶持资金投入专项资金池，撬动金融资金支持园区基础设施建设，收储土地1.2万亩以上，建设标准厂房50万平方米，确保新上项目容积率和投资强度，园区工业增加值增长8%。认真落实民营经济扶持政策，深入推进“两个10万元”微型企业创业培育工程，新增规上企业20户，确保民营经济增加值增长8%。

　　（五）加快城镇建设步伐

　　突出规划引领作用。科学谋划城市成长坐标，开展“多规合一”试点，充分发挥规划的龙头引导、空间管控和资源配置作用。启动新一轮城市总体规划修编，加强中心城区重点片区控规动态维护，完成美丽玉溪行动规划编制，提升完善美丽乡村、传统村落、重要交通沿线等区域村庄规划。开展全国城市设计试点工作，启动省级城市规划示范点、地下综合管廊等专项规划编制，完成城市色彩、建筑立面、景观照明等专项规划编制。加强规划实施监督管理，维护规划权威性，一张蓝图干到底。

　　抓好重点工程建设。统筹地上和地下基础设施建设，完成红塔大道地下综合管廊、中心城区雨污管网改造二期等工程建设，推进市规划展览馆、新黄路、火车西站站前广场和周边道路及地下综合管廊建设。初步建立城区安全骑行系统，健全城市休闲绿道网络。加快推进红星国际广场、临岸三千城等城市综合体建设，确保新天地商业广场正式运营。加快生活垃圾焚烧发电、中心城区再生水利用等项目进度，建设5座生活垃圾收集中转站。统筹县城和重点镇扩容提质，实施智慧小镇建设试点，启动8081户棚户区改造，推进老旧住宅小区综合整治，启动15个集镇“一水两污”项目和12个美丽乡镇建设。开展黑臭水体、公厕治理，新建改造城市公厕63座、旅游厕所50座。

　　提高城乡管理水平。全面开展“六城同创”，推进海绵城市建设，积极申报国家节水型城市。完成江川撤县设区后续工作，稳步推进通海撤县设市。推动户籍制度和居住证制度双落地，释放城市发展新动能。严格执行城市管理条例，加快“数字城管”建设，统筹政府、社会、市民三大主体，实施精细化、人性化管理，重点解决乱搭乱建、占道经营、交通拥堵等问题。全面推进建筑节能和绿色建筑工作，强化公共设施维修管护，搞好高仓、九龙池等5个城市重要节点“增绿添色”。推广“以克论净、深度保洁”经验，加强城市环境卫生综合整治，机械化清扫率达40%。

　　（六）着力改善生态环境

　　强化水污染防治。完成《云南省抚仙湖保护条例》修正。全面推进抚仙湖环境资源保护管理体制综合改革，完善统一托管措施办法，破除保护与发展的体制机制障碍。加快推进“四退三还”生态移民工程建设，建成抚仙湖北岸生态移民安置房一期和广南营、马房村搬迁地块湿地，加快北岸生态调蓄带二期建设，启动抚仙湖智慧生态环境监测系统及湖泊研究中心建设，建成抚仙湖展示中心。按照“一城五镇多村”规划布局，启动广龙小镇建设，打造特色旅游小镇和乡村。力争中央、省属企事业单位退出抚仙湖一级保护区取得实质性进展。抓好星云湖截污清淤、水体置换、水资源循环利用，推进杞麓湖沿湖农业减肥增效、村落污水收集处理及湿地公园建设。搞好东风水库董炳河流域综合整治和飞井海水库污染防治。

　　强化生态建设。编制玉溪市生态文明建设规划。完成营造林26.4万亩、退耕还林6万亩。加强森林资源保护管理，坚决遏制重大森林火灾发生。积极创建国家级生态乡镇和省级生态文明县。加强土壤环境保护和综合治理，促进退化、污染、损毁农田改良修复。加强地质、气象等灾害监测预警，搞好元江、曲江等河流水污染综合防治，治理水土流失面积190平方公里。

　　强化节能减排。开展重点能耗企业节能行动，搞好重点污染源实时在线监测，强化城镇污水处理厂建设运营管理，确保节能减排任务完成。加强环境影响评价审批工作监管指导，严格执行重点建设项目环评和“三同时”制度。坚持中心城区和县城PM2.5监测发布制度，加快燃煤锅炉“煤改气”，建成各县区机动车环保检测线，完成黄标车和老旧车年度淘汰任务，确保一级空气天数持续增加。

　　（七）全面深化各项改革

　　以经济体制改革为重点，激发市场活力、社会创造力和增长潜力。把“放、管、服”改革引向深入，推进行政审批事项承接、取消、下放和监管。整合产业类财政性涉企专项资金，改变投入方式，引导和扩大社会投资，促进基础设施建设和战略性新兴产业、高新技术产业、创新型企业发展。制定深化财税体制改革方案，健全全口径预算体系，加快国税、地税征管体制改革，提高税收征管质量效率。深化投融资体制改革，创新县城、重点镇基础设施建设投融资机制。深化农信社改革，发展壮大红塔银行，大力发展普惠金融，提高金融支持实体经济效率。推进机关事业单位养老保险制度改革，完成全市机关单位公务用车改革。加快商事制度改革，搞好“三证合一、一照一码”登记，推广全程电子化和电子营业执照应用。做好不动产统一登记工作。开展社会信用体系建设试点，打造信用玉溪。全面实施公立医院综合改革，扩大医疗保险付费制度改革试点范围，促进县乡村医疗服务一体化。统筹抓好教育、文化、科技等领域改革。落实中央深化司法体制和社会体制改革实施方案，不断提高社会治理能力。

　　（八）扎实推进社会建设

　　着力保障和改善民生。实施大学生创业引领“十百千万”计划、家庭服务企业“千户百强”工程，加快建设创业孵化平台，新增城镇就业2.4万人，7000名困难人员实现就业。实施全民参保登记计划，做好被征地农民养老保险工作，整合城乡居民基本医疗保险制度，加强社保基金管理，强化优抚对象帮扶，开展残疾人关爱行动，扩大社会救助覆盖面。完成20个居家养老服务中心、70个农村幸福院建设，改扩建5个乡镇敬老院，加快3个县社会福利服务中心建设。完成红塔区殡仪馆搬迁。继续办好10件惠民实事。

　　加快发展各项社会事业。加快推进“全面改薄”工程和学前教育第二轮3年行动计划，新建改造校舍13万平方米，完成美丽100校园行动计划暨校安工程和621所数字化校园建设；开工建设职教园区，积极申报建设滇西应用技术大学烟草学院。申报高新技术企业5户、创新型企业3户，新增院士工作站1个。启动建设广播影视传媒中心，申报李家山古墓群国家考古遗址公园，实施2个县级文化馆、4个乡镇文化站建设。加快市医院改扩建、市儿童医院等项目建设，完成2个乡镇卫生院、10个村卫生室标准化建设。贯彻落实全面两孩政策，分类实施无户口人员登记。广泛开展全民健身活动，办好各类体育赛事，搞好国家体育产业联系点工作。加强统计、外事侨务、保密、档案、史志等工作，加快妇女儿童、残疾人、红十字、关心下一代等事业发展。

着力创建平安玉溪。启动“七五”普法，完成村（居）民委员会换届选举工作。强化食品药品安全监管，严格落实安全生产责任制，深化重点行业领域安全整治。推进民族团结进步示范区建设，依法管理宗教事务。支持国防和军队建设，推动军民融合深度发展，争创第十届全国“双拥”模范城。强化信访维稳责任，完善社会治安防控体系，严打各类违法犯罪，加强反恐斗争，确保社会和谐稳定。

　　（九）强化政府自身建设

　　切实加强法治政府建设。认真做好地方立法工作。严格落实深入推进依法行政加快法治政府建设的实施意见，实行重大行政决策终身责任追究制度，严守重大行政决策程序，依法科学民主决策。坚决维护市委核心领导地位，严格执行市人大及其常委会的决议决定，自觉接受市人大法律监督和工作监督，接受市政协民主监督，认真听取人大代表、政协委员和民主党派、工商联、无党派人士、各人民团体的意见，扎实办理人大代表建议和政协提案，逐步提高解决率。推进行政执法规范化建设，严格规范公正文明执法。强化行政权力运行监督制约，全面推进政务公开。

　　切实加强效能政府建设。扎实开展“争先创优、跨越发展”大讨论大行动，确保玉溪各项工作干在实处、走在全省前列。持续巩固“三严三实”和“忠诚干净担当”专题教育成果，推进落实反“四风”常态化和长效化。加强学习培训，提高干部队伍深化改革、推动发展、维护稳定的能力。积极推进政府购买服务，推广电子政务和网上办事，提高政府服务便利化水平。健全完善重大决策部署监督检查机制，对有令不行、有禁不止、玩忽职守、为官不为的公职人员严督实查、严肃问责，确保政府及工作部门高效率运转、各项任务高质量完成。

　　切实加强廉洁政府建设。严格执行《中国共产党廉洁自律准则》和《中国共产党纪律处分条例》，落实党风廉政建设主体责任和监督责任，加强重点部门和岗位廉政风险防控。坚决落实中央八项规定精神和省市实施办法，严控“三公”经费支出。持续加强政风行风专项治理，坚决查处违纪违法案件，以零容忍的态度严惩腐败分子。

　　各位代表，实现经济社会发展“十三五”规划目标，既是历史责任，更是使命担当。让我们更加紧密地团结在以习近平同志为总书记的党中央周围，在中共玉溪市委的坚强领导下，以更严更实的作风攻坚克难，一步一个脚印砥砺前行，为在全省率先全面建成小康社会而努力奋斗！
